# Of puzzles and pavements: a quantitative exploration of leaf epidermal cell shape

**DOI:** 10.1111/nph.15461

**Published:** 2018-10-03

**Authors:** Róza V. Vőfély, Joseph Gallagher, Grace D. Pisano, Madelaine Bartlett, Siobhan A. Braybrook

**Affiliations:** ^1^ The Sainsbury Laboratory University of Cambridge Bateman Street Cambridge CB1 2LR UK; ^2^ Department of Biology University of Massachusetts 611 North Pleasant Street Amherst MA 01003‐9297 USA; ^3^ Department of Molecular, Cell and Developmental Biology University of California at Los Angeles 610 Charles E Young Dr. South Los Angeles CA 90095 USA; ^4^ Molecular Biology Institute University of California at Los Angeles 611 Charles E. Young Drive East Los Angeles CA 90095‐1570 USA

**Keywords:** cell shape, diversity, morphometrics, pavement cell, phylogeny

## Abstract

- Epidermal cells of leaves are diverse: tabular pavement cells, trichomes, and stomatal complexes. Pavement cells from the monocot *Zea mays* (maize) and the eudicot *Arabidopsis thaliana* (Arabidopsis) have highly undulate anticlinal walls. The molecular basis for generating these undulating margins has been extensively investigated in these species. This has led to two assumptions: first, that particular plant lineages are characterized by particular pavement cell shapes; and second, that undulatory cell shapes are common enough to be model shapes.
- To test these assumptions, we quantified pavement cell shape in epidermides from the leaves of 278 vascular plant taxa.
- We found that monocot pavement cells tended to have weakly undulating margins, fern cells had strongly undulating margins, and eudicot cells showed no particular undulation degree. Cells with highly undulating margins, like those of Arabidopsis and maize, were in the minority. We also found a trend towards more undulating cell margins on abaxial leaf surfaces; and that highly elongated leaves in ferns, monocots and gymnosperms tended to have highly elongated cells.
- Our results reveal the diversity of pavement cell shapes, and lays the quantitative groundwork for testing hypotheses about pavement cell form and function within a phylogenetic context.

## Introduction

The first cell was described by Robert Hooke in 1665; the empty cells of sectioned cork, seen under a microscope, were likened to the cells of a honeycomb (Hooke, 1665). Since that time, scientists have been observing plant cells in all of their diversity of form. The epidermal cells of leaves lend themselves readily to observation and display a great diversity of shapes and types: tabular pavement cells, complex trichomes, and stomatal complexes (Esau, 1977). Pavement cell shape, in particular, has been the focus of many recent studies probing the mechanistic basis of cell shape generation (Smith, 2003; Mathur, 2004; Fu *et al*., 2005; Panteris & Galatis, 2005; Kotzer & Wasteneys, 2006; Yang & Fu, 2007; Szymanski, 2009; Ivakov & Persson, 2013; Belteton *et al*., 2018).

Molecular studies of pavement cell shape generation have focussed almost exclusively on model genetic species such as Arabidopsis, maize, and *Oryza sativa* (rice) (Smith, 2003; Zhou *et al*., 2016; Belteton *et al*., 2018). All of these epidermides present dramatically undulating cell margins, while maize and rice (both grasses) also exhibit extreme cell elongation. From such studies a molecular framework for pavement cell shape generation has been proposed: margin undulation is a result of differential cell wall properties underlain by differential cytoskeletal patterning (Fu *et al*., 2005). Although intensely studied at a molecular level, and despite an early qualitative survey of leaf pavement cell shape (Linsbauer, 1930), it remains unclear how common margin undulation is across vascular plants.

Another abiding mystery is the biological reason (if any) for margin undulation – how does margin undulation impact organismal form and function? There are three long‐standing hypotheses in the field: (1) undulations may increase cell−cell contact between adjacent cells, allowing for more efficient chemical signalling (Galletti & Ingram, 2015); (2) undulating margins may increase epidermal integrity (think of a zipper) (Jacques *et al*., 2014); and (3) undulations may help leaves flex (Sotiriou *et al*., 2018). A fourth, more recent, hypothesis proposes that larger, isotropic, cells undulate to alleviate the stress caused by their own growth dynamics (Sapala *et al*., 2018). This cell‐strength hypothesis was put forth on the basis of observations in species closely related to Arabidopsis, and therefore represents a much‐needed foray into nonmodel species (Sapala *et al*., 2018). However, a phylogenetic context is an important consideration for any experimental designs of this type. Without taking phylogeny into account, one cannot be sure whether observed correlations are for functional reasons, or because of underlying relatedness of the species under study (Felsenstein, 1985). Quantitative assessments of cell shape, coupled to modern phylogenetic methods, allow for the disentanglement of contingency and functional relevance.

Here, we present a broad quantitative survey of epidermal pavement cell shape, analysed in an explicitly phylogenetic context. Utilizing morphometric methods, we determined two useful metrics for describing margin undulation (solidity) and base cell shape (aspect ratio, AR) across a wide swathe of the plant kingdom. We mapped solidity and AR values onto a phylogenetic tree of ferns and seed plants, and tested for phylogenetic signal. Phylogenetic signal assesses the propensity for trait values to be similar between closely related species. We found that cell shape was extremely diverse in our sample. While particular cell shape metrics characterized the ferns, gymnosperms, and monocots that we sampled, we could only detect phylogenetic signal at shallow phylogenetic levels in the eudicots. We conclude that a single underlying function for undulating cell margins is unlikely. We postulate that different optimizations may exist at shallow phylogenetic levels leading to the observable variation.

## Materials and Methods

### Sampling

Fully expanded adult leaves were collected from healthy plants grown in one of two locations between September 2015 and December 2017: The Botanic Garden of the University of Cambridge or the UMass Amherst Natural History Collection (see Supporting Information Table S1 for full species list). While cultivars and wild taxa were analysed together in this study, we employed a phylogenetically informed sampling strategy, being sure to sample broadly from all major clades of euphyllophytes.

### Sample preparation

Three methods of sample preparation were used. First, when possible, epidermal peels were taken from both sides of the leaf. When peels were unachievable, clear nail varnish was used to make impressions of epidermal cells, or a dissection and maceration protocol was followed. For dissection and maceration, roughly 5 × 5 mm asymmetric trapezoids were cut from the leaves, near the midrib, halfway along the length. The asymmetric shape allows keeping track of adaxial and abaxial sides through the several‐day‐long process. These pieces were placed in multiwell plates and soaked in  1 ml of a 1 : 7 mixture of acetic acid and 100% ethanol overnight at 4°C, stirred at 50 rpm. The following day, the solution was removed and samples were washed three times for 10 min. After the last wash, water was replaced by 1 ml of 1 M NaOH solution and left to stand for 24 h at room temperature, without stirring. Following this, the samples were washed again as before, and the solution was replaced by 1 ml of a solution containing 250 g chloral hydrate dissolved in 100 ml of a 1 : 2 mixture of glycerol and water. The samples remained in this solution for 3–5 d, until they became fully transparent. When the clearing finished, the samples were washed again as before and stored in water. Note that gaps in joint adaxial/abaxial sampling resulted from temporal shifts in methods as well as technical challenges of peeling in some cases.

### Staining and imaging

Samples were stained with 0.1% toluidine blue in water overnight, mounted on glass slides and covered with a coverslip. Images were acquired at ×100, ×200, ×400, ×700 or ×1000 magnification (depending on what was found appropriate for a given sample) using a Keyence VHX‐5000 digital microscope (Cambridge; Keyence UK Ltd, Milton Keynes, UK) or an Axioplan microscope (Amherst; Carl Zeiss Microscopy GmbH, Jena, Germany). Whenever possible, images were taken from both sides of the sample, at the same magnification. Images were saved in .tif format.

### Segmentation

Automatic segmentation of these images proved to be very difficult due to image defects on different length scales: dust grains, trichomes and hairs, uneven staining, varying light intensity across the image. Some of these difficulties can be eliminated by simpler image processing methods (filtering, smoothing) but others cannot. Therefore, we chose to perform segmentation manually, using a freely available image editor (gimp; Kimball & Mattis, 1996) on a tablet PC and tracing with a stylus, resulting in a black‐and‐white image of cells. The outlines of the cells were extracted in matlab (v.R2015b, Mathworks Inc., Natick, MA, USA) using basic built‐in functions. For each species, 30 cells were segmented per side (when both available), chosen to avoid underlying vasculature when possible.

### Leaf shape

To ensure an accurate representation of overall leaf shape, leaves or leaflets were flattened and scanned in front of a white background at a resolution of 300 dpi. These images were first binarized using an automatically determined simple threshold and the outlines were then extracted using matlab. One leaf sample (or leaflet for compound leaves) per species was used, the same leaf from which cells were extracted.

### Shape processing and statistical analysis

Cell outlines were used to calculate traditional morphometric descriptors (absolute area in μm^2^ for cells and mm^2^ for leaves, aspect ratio, circularity and solidity) and to extract the elliptic Fourier composition. Calculations were done using the momocs (Bonhomme *et al*., 2014) package in R. Aspect ratio was calculated by taking the ratio of each cell outline's width to its length (see later Fig. 2; coo_width/coo_length). Circularity (coo_circularitynorm) was defined as: perimeter squared divided by area, normalised by 4π. Solidity was calculated by dividing the area of a shape by the area of its convex hull (see later Fig. 2; coo_solidity).

For the Fourier analysis of cell shapes, cell outlines were aligned along their longest radii. We utilized 20 harmonics for the analysis, based on a cumulative harmonic sum >99.9% and test fitting outlines with undulating margins (Fig. S1). A normalized elliptical Fourier analysis was performed using momocs (efourier_norm, for area). Here, the Fourier coefficients are normalized according to the values describing the first harmonic. This normalization was included because randomly sampled species, when stacked, exhibited clean alignments without rotational artefacts.

Principal component analysis (PCA) was performed on the full dataset, again utilizing the momocs package. Data from all cells were used in PCA presented here, not means or representative cells. For cells, PCA was conducted using all cells from all species. For leaf shape, the outline from one leaf (or leaflet for compound leaves) per species was used to calculate traditional morphometric descriptors as above. Correlations between traditional metrics (Spearman's rho) were examined in R. *P*‐values associated with Spearman's rho were calculated using algorithm AS 89 (Best & Roberts, 1975; as implemented in R) to approximate the test statistic S. S was evaluated using Student's *t*‐test, with *n*‐2 degrees of freedom (as implemented in R).

### Tests for phylogenetic signal

The data matrix for phylogenetic analysis was constructed by extracting sequences from the matrix used for inferring a recent megaphylogeny of vascular plants (Zanne *et al*., 2014). When there was not an exact match for the species we sampled, we selected another species in the same genus from the megaphylogeny matrix. When there was no genus match, we retrieved sequences for each of the missing species from GenBank. The megaphylogeny matrix includes seven gene regions. We aligned each of these gene regions individually using mafft, as implemented in geneious, and concatenated each of these regions into a single matrix. A constraint tree, including all taxa in our analysis, was extracted from a second megaphylogeny using phylomatic (Webb & Donoghue, 2005; Slik *et al*., 2018). We used a constraint tree because we were not trying to infer phylogenetic relationships, but instead needed to generate a tree (with branch lengths) that showed established relationships that we could use in downstream analyses. Model and partitioning scheme selection was performed using partitionfinder (Lanfear *et al*., 2012). We analyzed our data matrix under the maximum‐likelihood information criterion using RAxML, as implemented on the CIPRES webserver (Miller *et al*., 2010; Stamatakis, 2014). The final data matrix, ML bootstrap tree, and constraint tree are all included in the Dryad data package (Vofely *et al*., 2018).

The resulting phylogeny was used in tests for phylogenetic signal using the R package phylosignal (Keck *et al*., 2016). Phylogenetic signal is the tendency of traits in related species to resemble each other more than in species drawn at random from the same tree. In a test for phylogenetic signal, the null hypothesis is that the values of a particular trait are distributed independently from their phylogenetic distance in a tree. There are several tests for phylogenetic signal. We selected local Moran's I test, which is designed to detect local hotspots of positive and negative trait autocorrelation (Münkemüller *et al*., 2012; Keck *et al*., 2016). The phylogeny figure was generated using the R package ggtree (Yu *et al*., 2017), with final editing performed in Illustrator (Adobe).

## Results

### Most taxa in our sample had slightly elliptical pavement cells with weakly undulating margins

**Figure 1:**
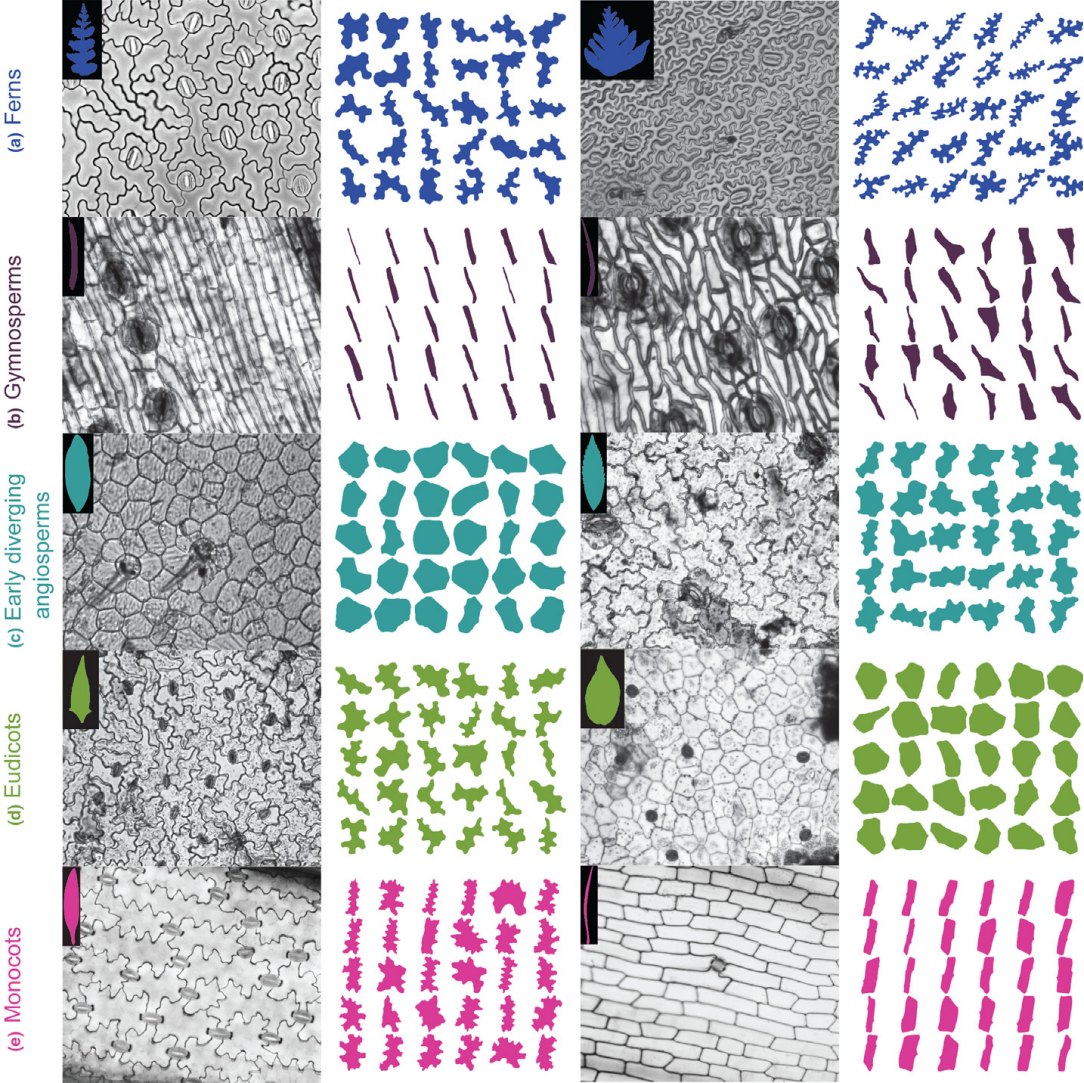
Epidermal peel images, leaf or leaflet outlines, and segmented cells for a small subset of sampled taxa. (a) *Microsorum pteropus* (Polypodiaceae), abaxial cells (left); and *Tectaria pseudosinuata* (Tectariaceae), abaxial cells (right). (b) *Araucaria* sp. (Araucariaceae), adaxial cells (left), and *Microcycas calocoma* (Zamiaceae), adaxial cells (right). (c) *Drimys winteri* (Winteraceae), adaxial cells (left); and *Chloranthus* sp. (Chloranthaceae), adaxial cells (right). (d) *Homalocladium platycladum* (Polygonaceae), adaxial cells (left); and *Catharanthus roseus* (Apocyanaceae), adaxial cells (right). (e) *Alstroemeria aurea* (Alstromeriaceae), adaxial cells (left); and *Hemerocallis fulva* (Xanthorrhoeaceae), adaxial cells (right). Leaf and cell outlines coloured according to major taxonomic divisions.

**Figure 2:**
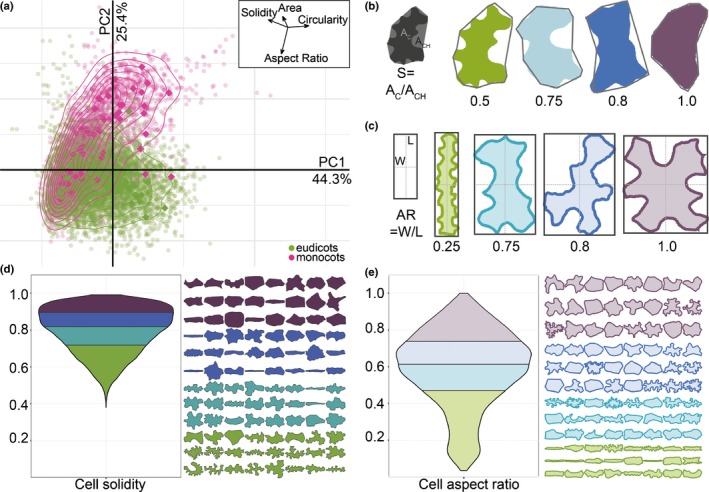
Traditional shape descriptors describe variation in base cell shape and margin undulation. (a) Principal component (PC) analysis of all epidermal cells sampled from monocots (pink) and eudicots (green) using traditional shape descriptors of aspect ratio (AR), area (A), circularity (C), and solidity (S). In this analysis, 69.7% of shape variance in the dataset was explained by the first two PC. The vectors describing the morphospace (inset) demonstrate how each shape descriptor relates to the first two components. (b) An illustration of cell solidity (S) calculated as the ratio of cell area to the convex hull area and its results from four representative cells with constant AR; colouring of representative cells matches quartiles bellow in (d). (c) An illustration of AR calculation as the ratio of maximal width to maximal length and its results from four representative cells with constant solidity value; colouring of representative cells matches quartiles bellow in (e). (d) The distribution of solidity values for our entire dataset, coloured according to quartiles. Twenty‐four cells from the median of each quartile are displayed with the same colour coding for reference. (e) The distribution of AR values for our entire dataset coloured according to quartiles. Twenty‐four cells from the median of each quartile are displayed with the same colour coding for reference.

To survey pavement cell shape across vascular plants, we sampled leaf epidermides from 278 vascular plant species, taking current phylogenetic hypotheses into account (Chase *et al*., 2016). A small sampling of our dataset is shown in Fig. 1 (full dataset (Vofely *et al*., 2018)). To quantify cell shape, we used the traditional shape descriptors of area, circularity, AR, and solidity (S) (Fig. 2; see the Materials and Methods section for definitions). We utilized these traditional metrics because we found that elliptical Fourier analysis did not perform well with our extremely diverse dataset (Fig. S2); elliptical Fourier analysis did a reasonable job of capturing aspect ratio variance but not margin undulations (Fig. S2). In a PCA with the traditional metrics, the sum of the principal component (PC)1 and PC2 together accounted for 69.7% of shape variance (Fig. 2a, monocots and eudicots as an example; Fig. S2, all clades). The vectors describing the traditional morphospace indicated that aspect ratio and solidity were strong perpendicular separators of cell shape (Fig. 2a, inset). Solidity was calculated by finding the area of the cell shape and dividing it by the area of the convex hull (Fig. 2b); the convex hull of an object can be conceived of as a rubber band stretched around the perimeter, so that in undulating cells the convex hull gaps away from the true perimeter (Fig. 2b). To calculate aspect ratio, cells were oriented according to their longest axis and the longest cell width was divided by the longest cell length in this orientation (Fig. 2c). Circularity represents how deviant a cell shape is from a perfect circle (Jacques *et al*., 2014; Wu *et al*., 2016) and captures both margin undulations and aspect ratios deviating from 1. This merged property was illustrated by the morphospace vector for circularity, which was the inverse sum of that for aspect ratio and solidity (Fig. 2a, inset). Therefore, we concluded that solidity and aspect ratio were good descriptors of margin undulation and base cell shape, respectively.

To determine whether pavement cells across vascular plants were characterized by a particular base cell shape or undulation pattern, we examined solidity and aspect ratio across our sampling. We found that most plant species displayed weak margin undulation. Solidity values for all species sampled occupied a range between 0.38 and 1, with a median of 0.802 (Fig. 2d; Table S1). This skew indicated that while most sampled pavement cells showed some degree of undulation, a minority of species sampled displayed complex margins (low solidity). Both Arabidopsis and maize pavement cells fell within the bottom 8% of solidity values for seed plants (*S*_At_ = 0.65, *S*_Zm_ = 0.63). The solidity metric is imperfect: curved cells with simple margins will also have a lower solidity value due to the calculation of convex hull area (Fig. 2b). In addition, solidity describes the deviation of the perimeter from the convex hull, but it does not provide information on the pattern of that deviation. For example, a margin might have a few deep lobes or many shallow lobes but have similar solidity values. This may have also been an advantage in our analysis: when the pattern of lobing was variable within a species (e.g. Arabidopsis), solidity would have been less sensitive to small variances in lobe number. Note that in a single species context, a new modification of Fourier analysis would prove an excellent tool to assess such variation (Sánchez‐Corrales *et al*., 2018). Our analyses of cell aspect ratio indicated that while most pavement cells were mildly elliptical in their base cell shape (median > 0.5); highly anisotropic or truly isotropic cells were rare in our data set (Fig. 2e). The distribution of aspect ratio across all species sampled occupied a range of 0.069–0.805 with a median value of 0.643 (Fig. 2e; Table S1). When we examined the variation in aspect ratio and solidity within each species, and compared it to the variation among all sampled cells it was clear that intraspecies variation was lower than interspecies variation (Fig. S3). This analysis also revealed a correlation between mean solidity and the variance in solidity: the more undulating the margin, the larger the standard deviation (Fig. S3). Based on all of the analyses so far, we concluded that the average epidermal cell in plants might best be represented by a slightly anisotropic cell with weak margin undulation.

### Patterns and diversity in pavement cell shape by vascular plant clade

**Figure 3:**
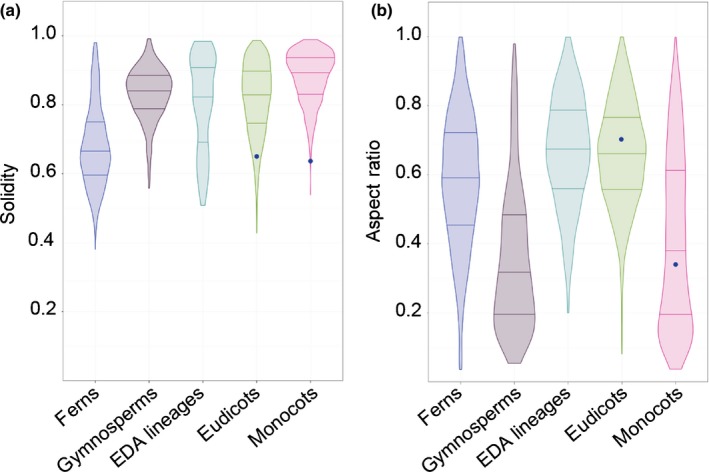
Solidity and aspect ratio distributions varied between clades. (a) Solidity (S) and (b) aspect ratio (AR) data are presented as distributions by clade for ferns, gymnosperms, early‐diverging angiosperm (EDA) lineages, monocots, and eudicots. Note that the mean S values for both Arabidopsis (*S*_At_ = 0.65) and maize (*S*_Zm_ = 0.64) fell within the tails of the eudicot and monocot distributions, respectively. By contrast, mean AR values for both Arabidopsis (AR_At_ = 0.71) and maize (AR_Zm_ = 0.33) lay close to the mean values for eudicots and monocots, respectively. Arabidopsis and maize mean AR and *S* values marked with blue dots. No sample size scaling has been applied.

To examine if trends in base cell shape and margin undulation might exist across the major clades of vascular plants with true leaves (megaphylls; Tomescu, 2009), we examined the distributions of aspect ratio and solidity in the following taxonomic groups: ferns, gymnosperms, the ANA grade, Chloranthales, magnoliids, monocots, and eudicots (Ruhfel *et al*., 2014; Chase *et al*., 2016; Schuettpelz *et al*., 2016). Here, we group the ANA grade (Amborellales, Nymphaeales, and Austrobaileyales), Chloranthales, and magnoliids using the term ‘early‐diverging angiosperm lineages’. We found that the ferns displayed a shift towards more undulate cells on average (Fig. 3a). Fern pavement cell margins have been described as more undulating than in the eudicots (Korn, 1976), an observation that held generally true in our data set. However, ferns exhibited the widest range of solidity values (0.38–0.98; Fig. 3a). The distribution of solidity values within the eudicots was also broad, although slightly less so than in ferns (Fig. 3a). Monocot and gymnosperm pavement cells tended to exhibit higher solidity values (less undulating margins) consistent with the qualitative literature for monocots (Linsbauer, 1930; Watson, 1942; Greguss, 1957; Stoddard, 1965; Ellis, 1976; Korn, 1976; Fig. 3a). With respect to aspect ratio, the ferns, early‐diverging angiosperms, and eudicots displayed normal distributions centring between 0.6 and 0.7, representing the slightly ellipsoidal base shape norm (Fig. 3b). In gymnosperms and monocots, the distributions were more skewed with medians below 0.4 indicating a trend towards a more anisotropic base shape in these groups (Fig. 3b). Taken together, these results indicate that pavement cells in the ferns, monocots, and gymnosperms share specific aspect ratio and solidity traits, while patterns of margin undulation have diversified in the eudicots.

**Figure 4:**
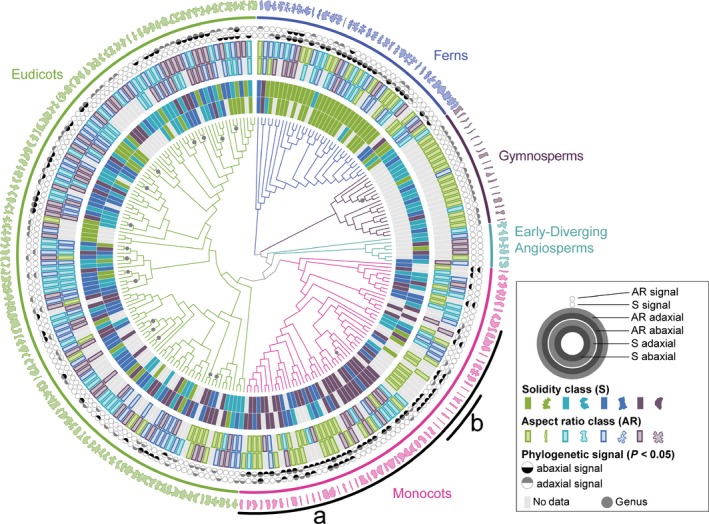
Pavement cells in the ferns, gymnosperms, and monocots were characterized by particular shape metrics. A maximum‐likelihood phylogenetic reconstruction of taxa sampled in our dataset (centre) surrounded by data rings depicting cell aspect ratio (AR) and solidity (S), and leaf AR for both adaxial and abaxial leaf surfaces (see key for positional key). Branch lengths are not shown in this figure, although they were used in all analyses. Taxonomic groups are indicated by colour. One representative cell shape from each species is depicted on the outermost ring. Evidence for phylogenetic signal was especially prevalent for cell metrics in the core monocots (a), and ferns (solidity); and in the grasses (b), and gymnosperms (AR). There was evidence for some phylogenetic signal at low taxonomic levels (family and/or genus) in the eudicots, but not across the clade as a whole. Each grey dot indicates multiple species in the same genus. All data and species names can be found in Supporting Information Table S1. The datamatrix and original treefiles are in the data repository (Vofely *et al*., 2018).

Our initial analysis did not take phylogeny into account, and cannot detect signal in specific orders or families obscured by considering, for example, ‘eudicots’ as a single group. To account for phylogenetic relationships, we mapped cell solidity and cell aspect ratio values onto a phylogeny of all the species that we sampled and tested for phylogenetic signal. Although related species tend to resemble one another, this is not true for every trait in every lineage. Tests for phylogenetic signal assess whether particular traits are more similar between closely related species than between distantly related species, or between species drawn at random from the same phylogenetic tree (Münkemüller *et al*., 2012; Kamilar & Cooper, 2013). Most of the ferns we sampled (*n* = 31/35, 89%) showed evidence for phylogenetic signal for solidity, with more complex cell margins (low solidity, Fig. 4a). In contrast, most core monocots (*n* = 38/46, 82%) have cells with less complex margins, falling within the first two quartiles of solidity (values closer to 1; Fig. 4b). Similarly, many core monocots had a strong signal for highly anisotropic cells (low aspect ratio, *n* = 23/35, 66%). This was especially pronounced in the grasses, where we found evidence for phylogenetic signal for cell aspect ratio in seven out of eight (88%) sampled grasses (Fig. 4c). The gymnosperms also exhibited phylogenetic signal for aspect ratio (Fig. 4d). In the eudicots, while there was some evidence for phylogenetic signal in aspect ratio signal, phylogenetic signal for solidity was concentrated in closely related species. There was no strong evidence for particular solidity values characterizing families, orders, or other major eudicot clades. Thus, while eudicot epidermal cell shapes were not distinguished by particular solidity values, fern epidermal cells are characterized by high undulation, and core monocot epidermal cells by low undulation.

### Abaxial leaf surfaces present more undulate cells

**Figure 5:**
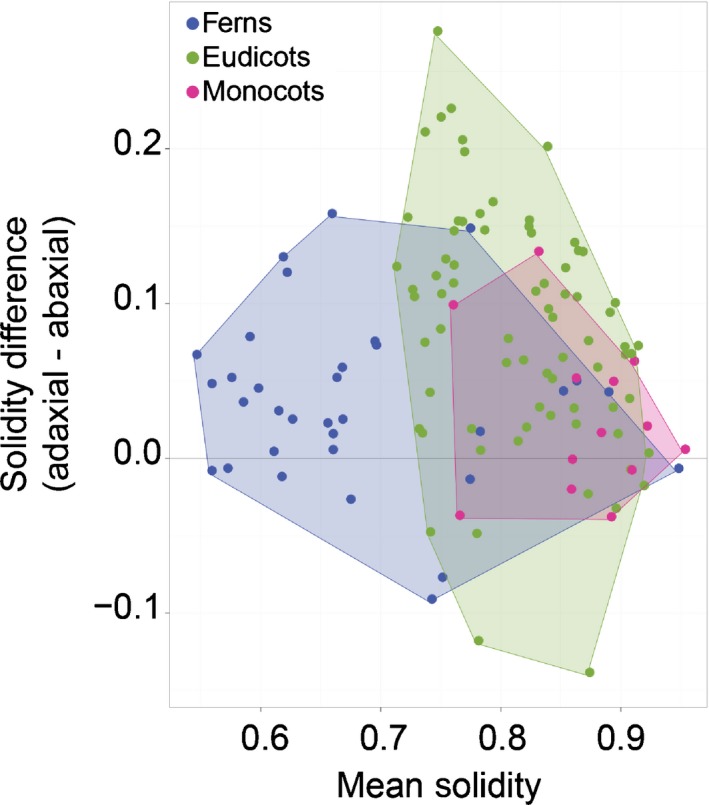
Cells on abaxial leaf surfaces tended to have more undulating margins. When there was a difference in mean cell solidity between the adaxial and abaxial leaf surfaces, solidity was often lower (higher undulation) on the abaxial leaf face. All data can be found in Supporting Information Table S1.

Sparse qualitative observations indicated that abaxial cells tend to have more undulate margins than adaxial cells (Linsbauer, 1930; Watson, 1942; Arogundade & Adedeji, 2017). To test this across our sampling, we calculated the difference between the average adaxial solidity and the average abaxial solidity in the 146 species for which we had data from both sides of the leaf (81 eudicots, 30 monocots, 28 ferns; see the Materials and Methods section). We found that when a difference in cell solidity was present, the abaxial cells tended to have more undulations (lower solidity, Fig. 5a), in line with the qualitative literature (Linsbauer, 1930; Watson, 1942; Arogundade & Adedeji, 2017).

### Anisotropic leaves tended to have anisotropic cells

We next wanted to explore connections between leaf shape and cell shape. Final epidermal cell shape over the surface of a leaf is a record of the developmental history of growth patterns; highly anisotropic cells indicate directional cell expansion, while regions of smaller cells indicate cell expansion coupled with division (Kaplan & Hagemann, 1991; Elsner *et al*., 2012). Leaf form is probably generated by complex growth patterns that we would be unable to detect with our sampling (Tsukaya, 2005; Kuchen *et al*., 2012; Vlad *et al*., 2014). However, in the Brassicaceae, a connection between growth direction, cell shape, and organ shape has recently been proposed (Sapala *et al*., 2018). In addition, in the flowers of *Saltugilia* spp. (Landis *et al*., 2015a,b) and *Mimulus lewisii* (Ding *et al*., 2017), highly anisotropic epidermal cells are present on anisotropic floral tubes, and more isotropic cells on petal lobes. We wondered whether we would be able to detect similar connections between cell shape and leaf shape at the broad scale of our dataset.

**Figure 6:**
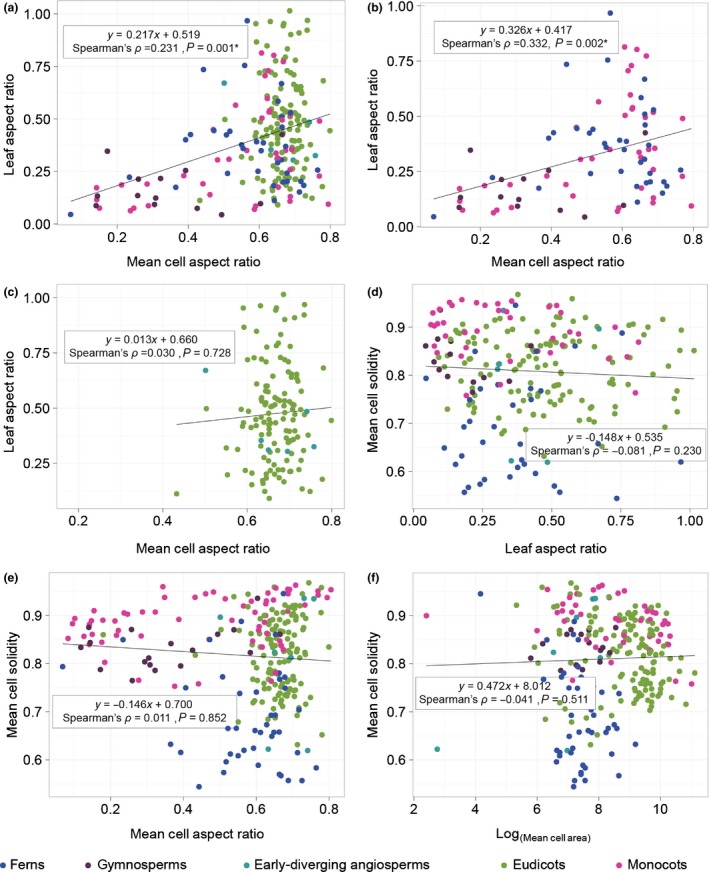
Anisotropic leaves tended to have anisotropic cells, but not in the eudicots or early‐diverging angiosperms. In the full dataset (a) and, in the ferns, gymnosperms and monocots (b), mean cell aspect ratio and mean leaf aspect ratio were correlated (regardless of leaf side). This correlation was not evident in the eudicots and early‐diverging angiosperms (c). No correlations were detected between leaf aspect ratio and mean cell solidity (d); mean cell aspect ratio and mean cell solidity (e); or log (mean cell area) and mean cell solidity (f). Linear regression lines are shown, along with Spearman's rho (ρ) correlation coefficients. All data can be found in Supporting Information Table S1.

To explore any connection between leaf and cell aspect ratio in our sampling, we examined correlations between leaf aspect ratio and cell aspect ratio. We found that highly anisotropic leaves tended to have highly anisotropic cells in the ferns, gymnosperms and monocots (Spearman's rho = 0.331, *P* = 0.002), but not in the eudicots and early‐diverging angiosperms (Spearman's rho = 0.030, *P* = 0.728; Fig. 6). This finding indicates that, in the anisotropic leaves of some vascular plants, anisotropic growth is likely to involve cell expansion, with very little coincident cell division, across large regions of the growing leaf. Were the cells to divide as they expanded, this connection between cell and leaf anisotropy would have been lost. By contrast, even in eudicots with leaves in the lowest aspect ratio quartile, cell aspect ratio levels never reached the lowest quartile. For example, in *Plantago afra* (eudicot) and *Hemerocallis fulva* (monocot), leaf aspect ratio values were similar (0.091 and 0.087, respectively. Both class 4), but mean cell aspect ratio values were not (mean AR_Pa_ *=* 0.67, class 3; mean AR_Hf_ = 0.17, class 1). Unlike in the ferns, gymnosperms and monocots, cell and leaf anisotropy were not correlated in the eudicots or early‐diverging angiosperms.

A second connection between cell shape and organ form that has been proposed is that the highly undulating cells characteristic of some eudicots are a consequence of cell expansion in all directions in the plane of the leaf lamina (Avery, 1933; Glover, 2000; Sapala *et al*., 2018). In this case, one would expect large cells to have low solidity (more undulations); or highly anisotropic leaves to have cells with high solidity values (fewer undulations); or that highly anisotropic cells would have high solidity values (fewer undulations). We detected no correlations between cell area and cell solidity; between leaf aspect ratio and cell solidity; or between cell aspect ratio and cell solidity (Fig. 6). Thus, margin undulation may not be related to leaf shape in our broad sample.

## Discussion

Here, we sampled leaf epidermides from 278 taxa, spanning a broad swathe of vascular plant diversity (Fig. 1; Table S1). Aspect ratio and solidity proved to be useful metrics for quantifying pavement cell shape in this sample (Fig. 2). Using these metrics, we found that fern pavement cell margins tended to be highly undulate, pavement cell margins in the monocots tended to be only weakly undulate, and that gymnosperm pavement cells tended to have low aspect ratios. In the eudicots and the early‐diverging angiosperms, cells tended to be slightly anisotropic, but were not characterized by particular solidity values (Figs 3, 4). We found some evidence for broad‐scale correlations: abaxial cells tended to have more undulating cell margins (Fig. 5); and anisotropic leaves tended to have anisotropic cells, but not in the eudicots or early‐diverging angiosperms (Fig. 6). Our results highlight the striking diversity of pavement cell shapes across the euphyllophytes, and suggest the molecular mechanisms that generate cell shape have both been conserved in some clades, and diversified in others.

Highly undulate pavement cells like those of Arabidopsis and maize were not common in our sample (Figs 2d, 3). This finding indicates that our molecular models of shape generation require modulation to reflect the diversity observed in the plant kingdom. The current molecular model for undulation (or protrusion) formation in Arabidopsis has actin concentrated at positions of protrusion outgrowth and microtubule bundling and restricts growth across indentations. This role of actin in protrusion outgrowth is consistent in maize and rice (Smith, 2003; Zhou *et al*., 2016). Patterns of actin and microtubules in several other species with undulating cell wall margins are also consistent with this model, although microtubules are likely to have numerous roles in pavement cells (Panteris & Galatis, 2005; Belteton *et al*., 2018). Given the distant relationship between Arabidopsis, a core eudicot, and maize and rice, core monocots, this mechanism may be common to all eudicots and monocots. In Arabidopsis, the patterning of alternating actin/microtubule patches is set up by active RHO‐RELATED PROTEIN FROM PLANTS 2 (ROP2). Active ROP2 promotes ROP‐INTERACTIVE CRIB MOTIF‐CONTAINING PROTEIN4 (RIC4)‐mediated fine actin accumulation while suppressing RIC1‐mediated microtubule bundling (Yang & Fu, 2007). In a situation where protrusion number and depth vary quantitatively on a phenotypic continuum (Fig. 2e), it is possible that the alternating pattern of actin and microtubules (and their controlling RICs) may be distinct between different species. It is equally probable that the patterning is conserved but the wall components and modifiers differ, leading to different wall mechanics and growth.

Differential cytoskeletal patterning is also likely to lead to differential wall thickness and material composition, as recently shown in several species with undulating pavement cell margins (Sotiriou *et al*., 2018). Differential biochemistry and mechanics of the wall are likely contributors to cell shape formation in Arabidopsis (Majda *et al*., 2017). These differential material properties must also be considered when considering the ‘reason’ for undulation: the mechanical integrity of tissues during stretching may be important (Sotiriou *et al*., 2018). It has recently been proposed that undulation may help an individual cell deal with its geometrically imposed stresses as it gets isotropically larger (Sapala *et al*., 2018). A sampling of Brassicaceae species (*n* = 16) showed a positive correlation between cell area and cell ‘lobeyness’ (Sapala *et al*., 2018) (‘lobeyness’ was solidity calculated as a perimeter ratio, as opposed to an area ratio here). In line with this hypothesis, pavement cell margin undulation is used in reconstructing paleoclimates because larger shade leaves often have larger pavement cells with more undulate margins (Kürschner, 1997; Dunn *et al*., 2015). We tested for this correlation in our dataset, but found no correlation between mean cell area and mean solidity. Similarly, we found no evidence for other predicted correlations: leaf aspect ratio and cell solidity, and cell aspect ratio and cell solidity were uncorrelated in our sample (Fig. 6). An attractive hypothesis, based on the data presented here, would be that margin undulation has different functions in different species, genera and families; the answer to several functional optimization problems may be to undulate margins.

We found that fern cell margins tended to be more undulating, while monocot cell margins tended to be less undulating (Figs 3, 4). This finding suggests that, in the ferns and in the core monocots, aspects of either the cell margin patterning machinery (e.g. actin and microtubule dynamics), or wall material properties, are shared between members of each clade. In the eudicots, where cells are not characterized by particular solidity values, these cell shape‐generating mechanisms and cell wall properties may be more variable at large evolutionary distances. However, solidity values between closely related species (e.g. species in the same genus) were often correlated, even in the eudicots (Fig. 4). Indeed, epidermal cell traits can be used as characteristics in systematics studies (Davila & Clark, 1990; Barone Lumaga *et al*., 2015; Jooste *et al*., 2016). This highlights the critical importance of accounting for phylogeny when testing hypotheses on the function of particular epidermal cell shapes (Felsenstein, 1985). For example, particular epidermal cell shapes have been proposed to be important in drought tolerance, in focussing light onto the photosynthetic machinery, or in providing mechanical stability to the epidermis (Bone *et al*., 1985; Poulson & Vogelmann, 1990; Augustine *et al*., 2015). When these hypotheses are tested using multiple different species, it is important to remember that cell shapes may be similar between species not because of a particular function, but because of underlying phylogeny.

The margins of pavement cells on the abaxial leaf surface tended to be more undulating than those on the adaxial leaf surface (Fig. 5a). The cause of this difference is ripe for discovery. In many cases, different sides of the leaves experience different microclimates; undulation exhibits some environmental plasticity and therefore it is plausible that more undulation on abaxial surfaces could relate to local environmental influences (Askenasy, 1870; Anheisser, 1900; Brenner, 1900; Watson, 1942). In addition, abaxial versus adaxial developmental identity may contribute to differential undulation (Ambronn, 1884; Avery, 1933; Watson, 1942). The number of cells of other cell types on the abaxial surface, particularly increased stomatal number (Driscoll *et al*., 2006), could contribute to increased undulation through a packing adjustment. Lastly, it is possible that differential growth rates between the two sides may relate to differential undulation. However, such growth differences would need to be balanced to finally yield a flat leaf. In *curl* tomato mutants, whose curled leaves exhibit larger cells on the abaxial epidermis, there are no qualitative differences in abaxial (or adaxial) cell undulations from the wild‐type (Pulungan *et al*., 2018). This situation suggests that, in tomato, differential growth rates are not contributing to differential adaxial versus abaxial margin undulation. Here too, differential margin undulation between the leaf sides may have different causes in different plant taxa and under different circumstances.

We found evidence for a correlation between cell and leaf aspect ratio in the ferns, gymnosperms and monocots: anisotropic cells tended to occur in anisotropic leaves. By contrast, eudicots with highly anisotropic leaves did not have similarly anisotropic cells (Fig. 6c). This finding indicates that growth dynamics differ considerably between different species and different clades, even when leaf form is superficially similar (Gázquez & Beemster, 2017). While cell division and elongation are both essential drivers of growth and development, the development of plant form can only be understood by studying the balance between these two processes, and their regulation in an organ‐level and organismal context (Kaplan & Hagemann, 1991; Ranjan *et al*., 2015; Sablowski, 2016; Martinez *et al*., 2017). Further taxonomically broad exploration of cell expansion and division over time, similar to that applied in Arabidopsis (Andriankaja *et al*., 2012; Elsner *et al*., 2012; Rolland‐Lagan *et al*., 2014), would prove highly informative for understanding the breadth of organ growth mechanisms present in the plant kingdom.

### Conclusions

Our analysis has revealed striking diversity in leaf epidermal cell shape. This quantitative analysis has allowed for mapping of shape metrics in a phylogenetic context. This demonstrated that, while closely related eudicots tend to share cell shape characteristics, there is no global trend towards highly undulate margins in the eudicots. The lack of consistent highly undulating cell margins, like those observed in Arabidopsis, make a strong case for expansion beyond a single model system. Similarly, while maize epidermal cells have highly undulate margins, monocots tended to have weakly undulating cells, again pointing to a need to work in species beyond the grasses.

How might epidermal shape diversity arise? Based on the well resolved molecular network regulating cell shape in Arabidopis (and maize) (Smith, 2003; Mathur, 2005; Panteris & Galatis, 2005; Yang & Fu, 2007; Qian *et al*., 2009; Szymanski, 2009), an attractive hypothesis might be that the patterning system, centred on ROP‐mediated exclusivity between actin and microtubule position, is variable among species. Variability in the patterning of cell wall synthesis and modification would yield variation in cell undulation. Alternatively, the cytoskeletal patterning mechanism might be perfectly consistent in most species (suggested by conservation between Arabidopsis and maize), but cell wall synthesis and modification might differ between species and clades. Indeed, primary cell wall composition is highly variable across plants (Popper, 2008).

Our data and analysis indicate that undulation is a continuous trait that shows little conservation in the eudicots, a potentially puzzling outcome given the recent functional claims made in the literature (Sapala *et al*., 2018; Sotiriou *et al*., 2018). However, we do not find this unsettling. Instead it implies that in the evolutionary optimization of function, in which a plethora of needs and functions must be balanced, there are likely to be many different solutions to functional problems, and sometimes undulations count and sometimes they do not. For example, in species in which wall composition cannot be altered to compensate for cell area increases, the topological stress‐minimization function proposed (Sapala *et al*., 2018) might win, yielding undulating cells. In another case, in which leaves are subjected to extreme and repeated tensile stress, the material‐flexibility function proposed (Sotiriou *et al*., 2018) might result in undulating cells; two different functions with a similar result. What should be taken from our work here is that potential functional consequences of this form should be considered within a phylogenetic context. Indeed, exploration of form−function relationships with respect to pavement cell form would best be conducted at shallow phylogenetic levels and by looking at species that are closely related, with well resolved phylogenies (so that one can account for phylogeny) that differ ecologically, show diversity in cell shape, and are tractable model systems.

### Future prospects for the dataset

The wide sampling of leaf epidermides utilized in this study is freely available (Vofely *et al*., 2018). While this study focussed on pavement cell shape, the raw images contain information on other epidermal characters such as stomatal and trichome morphology and number (occasionally), cell shape diversity as it relates to leaf anatomy (e.g. influence of venation), and differences in abaxial and adaxial characteristics. Such a wealth of resources should be valuable to many researchers in plant science. The size of the dataset also make it suitable for machine learning analysis and the potential development of classification algorithms based on epidermal morphologies and would be useful for classification of extant and fossil specimens (Wilf *et al*., 2016).

## Author contributions

SAB conceived of the original project and designed experiments with RVV and MB MB and GDP devised a sampling strategy. RVV and GDP collected samples, prepared and imaged samples, generated outlines, and utilized momocs for analyses. RVV, JG, and MB performed in depth analyses with momocs, generated PCAs, and examined correlations between descriptors (SAB). MB performed phylogenetic signal analyses. SAB and MB wrote the manuscript and prepared figures, with assistance from RVV and JG.

## Supporting information

Please note: Wiley Blackwell are not responsible for the content or functionality of any Supporting Information supplied by the authors. Any queries (other than missing material) should be directed to the *New Phytologist* Central Office.

**Fig. S1** Harmonic assessment for elliptical Fourier analysis.
**Fig. S2** Results from traditional and elliptical Fourier analysis of cell shapes.
**Fig. S3** Examining variance in aspect ratio and solidity.

**Table S1** Data table with species information and mean morphometric values.

## Data Availability

The datasets generated during the current study, including the phylogenetic datamatrix and trees, have been deposited to Dryad (doi: 10.5061/dryad.g4q6pv3; (Vofely *et al*., 2018)). Mean shape metrics are included in Table S1. For some species, scanning electron micrographs are also available upon request, although not included in this study. Code for analyses and figure generation is available on github (https://github.com/BartlettLab/LeafEpidermis).
