# Supplementary material for: Of puzzles and pavements: a quantitative exploration of leaf epidermal cell shape
Source: New Phytol. 2018 Oct 3;221(1):540–52. doi: 10.1111/nph.15461 (PMC6585845; doi:10.1111/nph.15461)
Supplement: Supplementary file 1 — Fig. S1 Harmonic assessment for elliptical Fourier analysis. Fig. S2 Results from traditional and elliptical Fourier analysis of cell shapes. Fig. S3 Examining variance in aspect ratio and solidity. [file NPH-221-540-s001.pdf]

New Phytologist

***New Phytologist* Supporting Information**

Article title: Of puzzles and pavements: a quantitative exploration of leaf epidermal cell shape
Authors: Róza V. Vőfély, Joseph Gallagher, Grace D. Pisano, Madelaine Bartlett and Siobhan A. Braybrook
Article acceptance date: 21 August 2018

The following Supporting Information is available for this article:

**Fig. S1** Harmonic assessment for Elliptical Fourier Analysis.

**Fig. S2** Results from Traditional and Elliptical Fourier analysis of cell shapes.

**Fig. S3** Examining variance in aspect ratio and solidity.

**Table S1** Data table with species information and mean morphometric values.

**Fig. S1** Harmonic assessment for Elliptical Fourier Analysis. Representative cell shapes of solidity values 0.6 (a) and 0.4 (b) with fitted ellipses at several harmonic ranks. While the less undulatory cell (S=0.6) could be fit with 15 harmonics (red dashed box), the more undulatory (S=0.4) cell is best fit by 20 harmonics (red dashed box). For this reason, 20 harmonics were chosen for our analyses. (c) a graph of the cumulative sum harmonic power for our dataset (a measure of how many harmonics (h) are required to fit the data) and 99.9% is easily reached with h16. S, solidity.

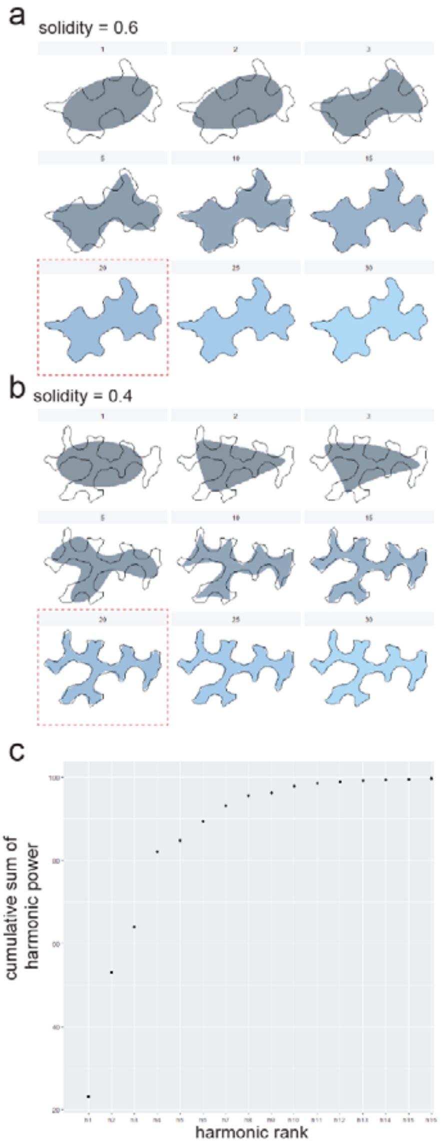

**Fig. S2** Results from Traditional and Elliptical Fourier analysis of cell shapes. Morphospace for PCA1 and PCA2 (a) Traditional; (b) Elliptical Fourier, split by clade. The analysis was performed on all groups together but shown here independently. Fourier analysis morphospaces for two monocot examples (c) *Danthonia californica* and (d) *Spathiphylium wallisii*, exhibiting margin undulation and cell anisotropy, respectively. Representative cells shapes (I), eigenvalues for PCA1 (purple) and PCA2 (turquoise) (II), and PCA1 and PCA2 morphospaces (III); this analyses demonstrates the utility of Elliptical Fourier analysis for cell base shape (anisotropy in *Danthonia* is well described by 88.7% in PCA1) and its lacking when margin undulation is the variable trait (*Spathiphyllum*, low percentage of variation explained by any one PCA as show in eigen values). PC, principal component; PCA, principal component analysis.

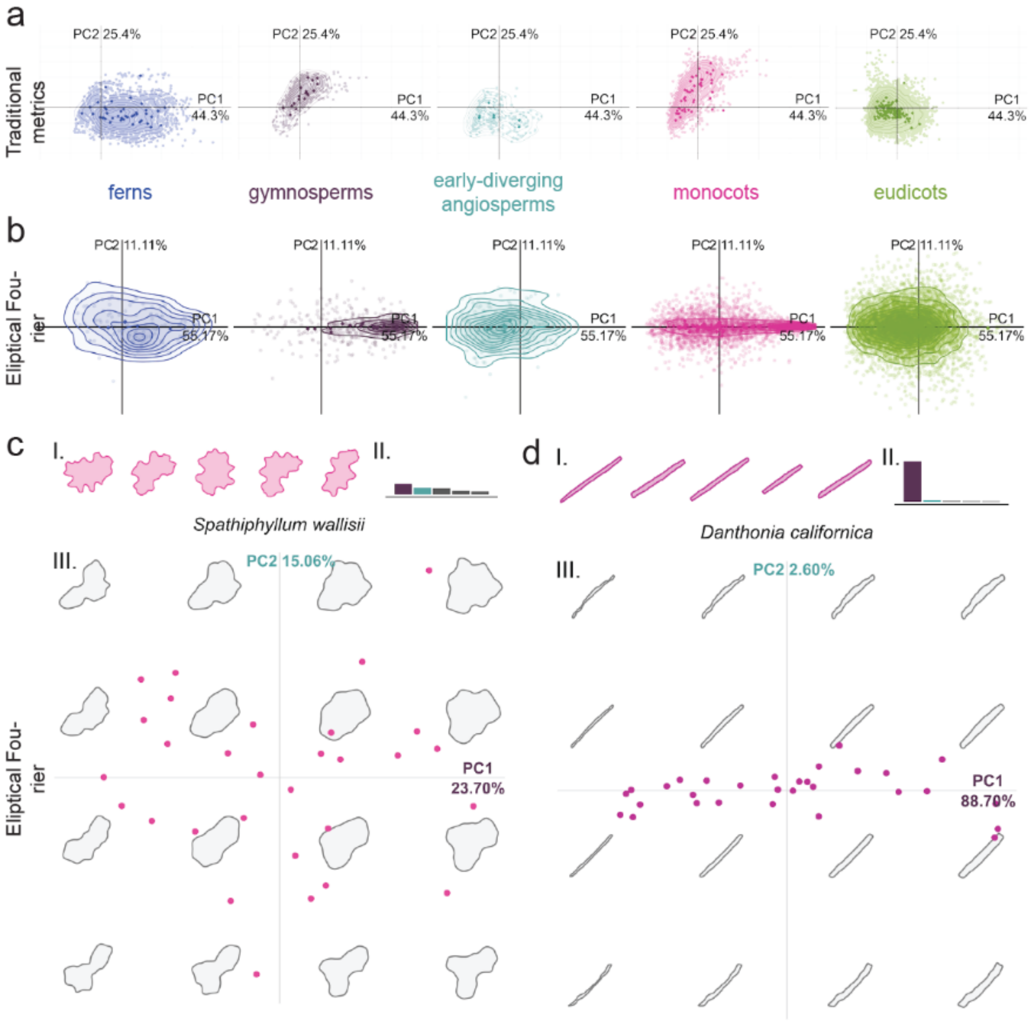

**Fig. S3** Examining variance in aspect ratio and solidity. (a) Box plots of the standard deviations, by species, for aspect ratio (AR) and solidity (S) demonstrate the amount of variance seen within species. 'X' marks the standard deviations for AR (purple) and S (pink) calculated for the entire data set demonstrating the variance within all cells sampled. For solidity, only 2 species had higher inter-species variance than the intra-species variance: *Acanthus spinosus* and *Sisymbrium austriacum*. Thirty-two (32) species had higher aspect ratio inter-species variance than that intra-species. (b) Mean solidity by species plotted against standard deviation in solidity by species, with a least-squares regression. The rho value and p-value indicate a weak but significant correlation between solidity mean and standard deviation, meaning the lower the solidity (more undulatory) the more variance, within a given species.

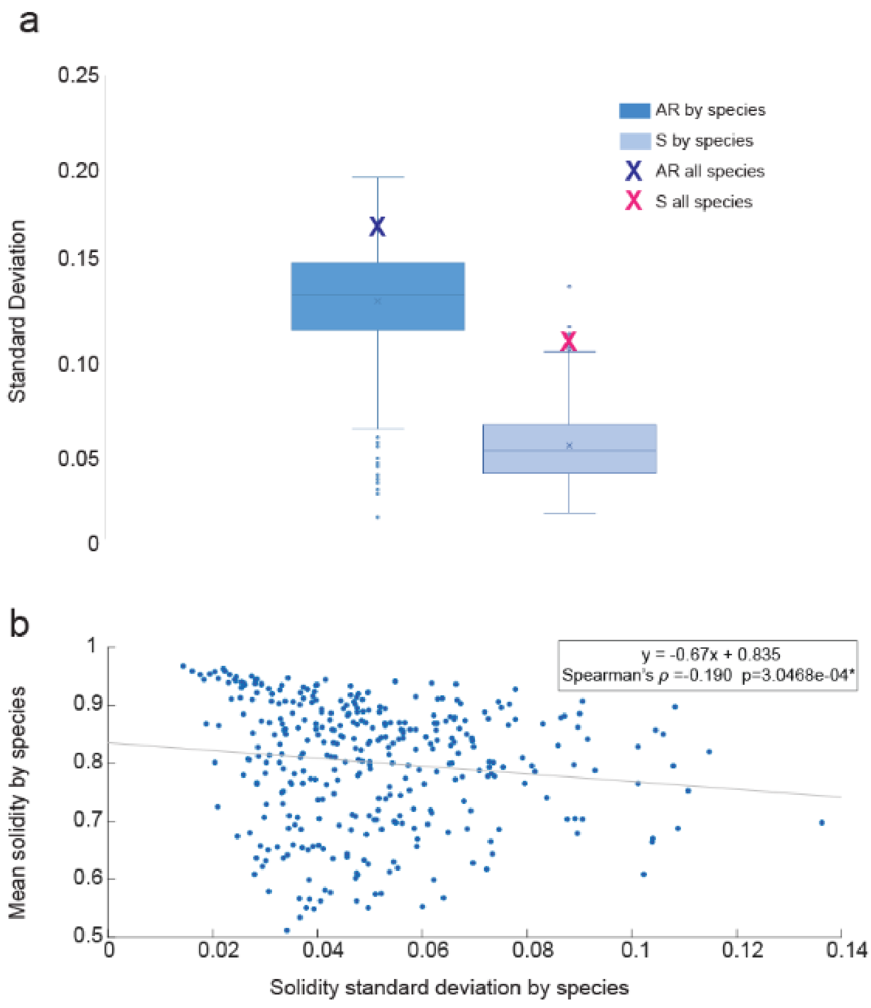

**Table S1** Data table with species names, species names used in phylogenetic analyses, order, family, and major group. Sample identification code is included for reference to the Dryad dataset (doi:10.5061/dryad.g4q6pv3). Traditional morphometric means and standard deviations are provided per species (per leaf side, when available). NA, not available.

*Available as separate download.*
